# Chest wall reconstruction using Mersilene mesh and titanium rib plates after extensive tumour resection with immediate postoperative extubation: a case report

**DOI:** 10.1093/jscr/rjag607

**Published:** 2026-08-14

**Authors:** Muhammad Azmi Agung, I Dewa Gede Nalendra, Dhihintia Jiwangga Suta Winarno, I Gusti Bagus Chandogya

**Affiliations:** Residency Program, Department of Thoracic, Cardiac, and Vascular Surgery, Faculty of Medicine, Universitas Airlangga, Surabaya 60131, Indonesia; Department of Thoracic, Cardiac, and Vascular Surgery, Dr. Ramelan Navy Hospital, Surabaya 60244, Indonesia; Department of Thoracic, Cardiac, and Vascular Surgery, Dr. Soetomo General Academic Hospital, Surabaya 60286, Indonesia; Department of Thoracic, Cardiac, and Vascular Surgery, Dr. Soetomo General Academic Hospital, Surabaya 60286, Indonesia

**Keywords:** chest wall tumor, adenocarcinoma, chest wall reconstruction, Mersilene mesh, rib plate, en bloc resection

## Abstract

A 36-year-old male presented with a progressively enlarging right anterior chest wall mass over one year. Preoperative biopsy revealed adenocarcinoma, with no definite primary tumor identified on thoracic imaging or evaluation of adjacent organs. Computed tomography demonstrated a large heterogeneous mass with rib destruction and extension into the surrounding soft tissue and pleura. The patient underwent en bloc resection of the involved chest wall, including the third to sixth ribs, followed by reconstruction using Mersilene mesh and titanium rib plates. The combined reconstruction provided soft tissue coverage, prevented intrathoracic herniation, and restored thoracic skeletal stability. Immediate postoperative extubation was achieved without paradoxical chest wall movement, flail chest, or respiratory compromise. This case highlights that combined mesh reinforcement and rigid fixation may support early thoracic stability after extensive chest wall resection. At the 6-month follow-up, the patient remained clinically stable without respiratory compromise, paradoxical chest wall movement, wound infection, prosthesis exposure, or clinically evident implant-related complication.

## Introduction

Resection of chest wall tumors involving multiple ribs may disrupt thoracic stability and impair postoperative ventilation. Loss of structural continuity can result in paradoxical motion, making reconstruction essential to restore mechanical integrity and respiratory function. Achieving adequate oncological resection remains the cornerstone of treatment, although extensive defects require careful integration of oncological and reconstructive strategies. Adenocarcinoma involving the chest wall is uncommon and often raises concern for metastatic disease or direct invasion from adjacent structures such as the lung and pleura [1].

Various reconstructive approaches have been described. Synthetic mesh provides soft tissue support, whereas rigid fixation systems restore skeletal stability [2]. Titanium-based rigid fixation has gained increasing attention for large chest wall defects because it may restore skeletal continuity, maintain thoracic contour, and reduce paradoxical motion after extensive resection. However, implant-related complications, including wound infection, plate fracture, and prosthesis exposure, remain important considerations [3–5]. This case highlights immediate postoperative extubation as a practical clinical indicator of adequate thoracic stability after combined mesh and titanium rib plate reconstruction.

## Case report

A 36-year-old male presented with a progressively enlarging right anterior chest wall mass for 12 months. The lesion was painless and was not associated with dyspnea, cough, fever, or weight loss. Physical examination revealed a firm, non-tender mass without overlying skin changes. Histopathological evaluation from a prior biopsy confirmed adenocarcinoma.

**Figure 1:**
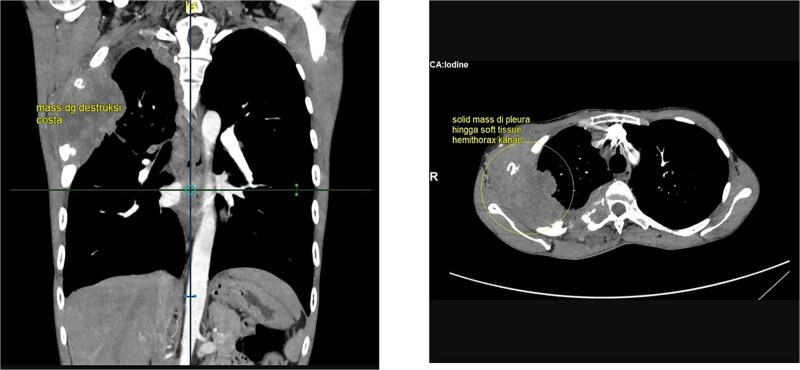
Contrast-enhanced CT scan of the thorax. Coronal and axial images reveal a large, heterogeneous mass occupying the right hemithorax, arising from the chest wall and extending into the pleura and adjacent soft tissues. The lesion demonstrates rib destruction, consistent with locally aggressive disease. Areas of internal low attenuation suggest necrosis. The mass abuts and partially invades the adjacent lung parenchyma, with irregular and spiculated margins.

Contrast-enhanced computed tomography (CT) demonstrated a heterogeneous mass measuring 14.7 × 9.6 × 6.9 cm with central necrosis, involving the right upper chest wall. The tumor infiltrated the subcutaneous tissue, intercostal muscles, and pleura, with destruction of the third to sixth ribs. Extension into the adjacent lung parenchyma with spiculated margins was observed. Suspicious lymphadenopathy was present in the right axillary and peritumoral regions. Multiple contralateral lung consolidations raised concern for metastatic disease. Differential diagnoses included metastatic adenocarcinoma of unknown primary, primary chest wall malignancy, and locally invasive primary lung carcinoma (Fig. 1).

Given the extent of osseous destruction and soft tissue involvement, the patient underwent en bloc resection with the aim of achieving complete oncological resection. Resection included the third to sixth ribs and involved soft tissues. Mersilene mesh was used to provide soft tissue support and prevent intrathoracic herniation, while titanium rib plates were applied to restore structural continuity of the thoracic cage. This combined strategy was selected because large defects involving multiple ribs require both soft tissue reinforcement and rigid skeletal stabilization to maintain respiratory mechanics [3, 4, 6, 7].

**Figure 2:**
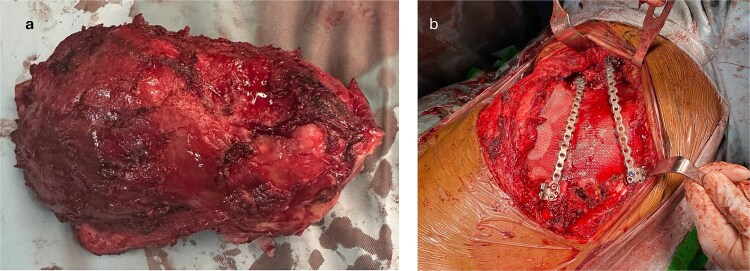
Surgical specimen and chest wall reconstruction. (a) Gross specimen of the resected chest wall tumour after en bloc resection, demonstrating an irregular and heterogeneous mass. (b) Intraoperative reconstruction of the chest wall defect using titanium rib plates for skeletal stabilization and Mersilene mesh for soft tissue coverage.

Final histopathological examination confirmed adenocarcinoma involving the chest wall. Further immunohistochemical evaluation and multidisciplinary oncological assessment were planned to determine the most likely primary origin. The patient was extubated immediately after surgery without paradoxical chest movement or respiratory compromise. Postoperative recovery was uneventful, and the patient was referred for consideration of adjuvant therapy. At the 6-month follow-up, the patient remained clinically stable, with no respiratory compromise, paradoxical chest wall movement, wound infection, prosthesis exposure, or clinically evident implant-related complication. He continued multidisciplinary oncological follow-up for further evaluation and adjuvant treatment planning. The surgical specimen and reconstruction are shown in Fig. 2.

## Discussion

Extensive chest wall resection involving multiple ribs creates a risk of postoperative respiratory impairment due to disruption of thoracic cage stability. This risk is particularly relevant in anterior defects, where loss of structural continuity may lead to paradoxical movement and compromised ventilation. Reconstruction should therefore address both soft tissue coverage and skeletal stability.

Synthetic mesh materials are widely used to cover full-thickness defects and protect intrathoracic organs [7]. However, mesh alone may not provide sufficient rigidity when multiple ribs are resected. Clinical series have shown that large defects often require additional structural support to maintain respiratory mechanics [6]. Titanium-based systems may restore chest wall contour and mechanical continuity, but their use must be balanced against implant-related morbidity [3–5].

In this case, Mersilene mesh and titanium rib plates were combined to address both components of the defect. Mesh provided soft tissue coverage, while rigid fixation restored mechanical continuity of the thoracic cage. Immediate postoperative extubation without respiratory compromise suggests that adequate early stabilization was achieved. This observation is consistent with contemporary reports showing that titanium-based reconstruction may facilitate early respiratory stability. Clermidy *et al.* [4] reported that most patients undergoing reconstruction with titanium bars and sternal plates were extubated on the day of surgery, with low rates of paradoxical chest movement. Similarly, recent experience with titanium mesh demonstrated preservation of chest wall volume and respiratory mechanics without early organ herniation or mesh failure [8].

Nevertheless, rigid reconstruction is not free from risk. Bergovec *et al.* [5] reported substantial complication rates after titanium plate reconstruction, including wound complications and implant-related failure. Therefore, long-term follow-up remains necessary to evaluate implant integrity, wound healing, local recurrence, and delayed respiratory complications. Despite these limitations, this case supports the concept that reconstructive planning should aim not only to restore anatomical integrity but also to support early postoperative respiratory function.

## Data Availability

The data supporting the findings of this case report are available from the corresponding author upon reasonable request.
